# Trait coordination, mechanical behaviour and growth form plasticity of *Amborella trichopoda* under variation in canopy openness

**DOI:** 10.1093/aobpla/plw068

**Published:** 2016-11-11

**Authors:** Santiago Trueba, Sandrine Isnard, Daniel Barthélémy, Mark E. Olson

**Affiliations:** 1 IRD, UMR AMAP, Laboratoire de Botanique et d’Écologie Végétale Appliquées, Noumea, BPA5, 98800, New Caledonia; 2 CIRAD, BIOS Direction, and INRA, UMR AMAP, F-34398 Montpellier, France; 3 Instituto de Biología, Universidad Nacional Autónoma de México, Tercer Circuito s/n de Ciudad Universitaria México, México DF 04510, México

**Keywords:** Adaptation, allometry, biomechanics, leaf mass per area, light environment, modulus of elasticity, phenotypic plasticity, plant architecture

## Abstract

Understanding the distribution of traits across the angiosperm phylogeny helps map the nested hierarchy of features that characterize key nodes. Finding that *Amborella* is sister to the rest of the angiosperms has raised the question of whether it shares certain key functional trait characteristics, and plastic responses apparently widespread within the angiosperms at large. With this in mind, we test the hypothesis that local canopy openness induces plastic responses. We used this variation in morphological and functional traits to estimate the pervasiveness of trait scaling and leaf and stem economics. We studied the architecture of *Amborella* and how it varies under different degrees of canopy openness. We analyzed the coordination of 12 leaf and stem structural and functional traits, and the association of this covariation with differing morphologies. The *Amborella* habit is made up of a series of sympodial modules that vary in size and branching pattern under different canopy openness. *Amborella* stems vary from self-supporting to semi-scandent. Changes in stem elongation and leaf size in *Amborella* produce distinct morphologies under different light environments. Correlations were found between most leaf and stem functional traits. Stem tissue rigidity decreased with increasing canopy openness. Despite substantial modulation of leaf size and leaf mass per area by light availability, branches in different light environments had similar leaf area-stem size scaling. The sympodial growth observed in *Amborella* could point to an angiosperm synapomorphy. Our study provides evidence of intraspecific coordination between leaf and stem economic spectra. Trait variation along these spectra is likely adaptive under different light environments and is consistent with these plastic responses having been present in the angiosperm common ancestor.

## Abstract (precis)

Finding that Amborella trichopoda is sister to the rest of the angiosperms has raised the question of whether it shares certain key functional trait characteristics and plastic responses apparently widespread within the angiosperms at large. With this in mind, Trueba et al. tested the hypothesis that local canopy openness induces plastic responses in Amborella. The authors provide evidence of intraspecific coordination between leaf and stem economic spectra in this key species. Moreover, by presenting the first architectural and biomechanical characterization of Amborella, their study offers new insights for the understanding of the early sequences of angiosperm form evolution.

## Introduction

Comparative biology is built on an understanding of the patterns of distribution of organismal characters. Those that uniquely characterize single clades are known as synapomorphies. The nested hierarchy of synapomorphies across the tree of life helps to reconstruct the patterns of relationships between taxa (Nixon and Wheeler 1990). In contrast, characters that arise repeatedly can reflect an array of processes from convergent evolution to shared propensities for evolving similar traits independently, reflecting convergence or parallelism (Harvey and Pagel 1991; Losos 2011; Scotland 2011). To understand the distribution of traits within a group, it is crucial to study as wide an array of lineages as possible. Among the flowering plants, *Amborella trichopoda* (Amborellaceae), a dioecious woody plant endemic to the moist forests of New Caledonia, has attracted the attention of plant science since the end of the last century, after several phylogenetic studies supported the position of *Amborella* as the single surviving representative of a lineage sister to all other extant angiosperms (Mathews and Donoghue 1999; Soltis *et al.* 1999; Mathews and Donoghue 2000; Qiu *et al.* 2000; Soltis *et al.* 2000; Amborella Genome Project 2013; Poncet *et al.* 2013).

Because of its phylogenetic position, the analysis of *Amborella* traits is of interest in understanding the evolution of the ecology, function and structure of flowering plants (Amborella Genome Project 2013). Finding that there are features shared by *Amborella* and other flowering plants, but not the gymnosperms, could point to angiosperm synapomorphies or important convergent features. On the other hand, finding that there are features shared by *Amborella* and the gymnosperms but not the rest of the angiosperms could reveal useful information regarding the early sequence of character evolution within the flowering plants. This study focuses on patterns, potentially synapomorphic and homoplasious, currently being documented all across the woody plants. Through the description of *Amborella*'s architectural and biomechanical organization, combined with analyses of the coordination of functional leaf and stem traits and their variation under different light environments, we can provide elements for understanding the evolution of growth forms in the flowering plants and how these forms vary developmentally under different light conditions.

One of the longstanding questions in the study of angiosperm structure concerns the habit and growth form of the earliest flowering plants. *Amborella* has a multi-stemmed habit with stems that have been described as semiscandent (Feild *et al.* 2001; Feild and Wilson 2012). This growth habit is often called ‘cane-like’ and seems to be widespread in the ‘basal’ lineages of angiosperms (e.g. *Aristolochia*, *Eupomatia*, *Illicium*, *Piper*, *Sarcandra*, *Thottea* and *Trimenia*) (Carlquist 1996; 2001; Feild and Arens 2005; Carlquist 2009; Isnard *et al.* 2012), pointing to a potential angiosperm synapomorphy. The cane-like habit seems to be characterized by a combination of sympodial growth and mechanical laxness, with stems that are relatively long for their tissue stiffnesses (Feild and Arens 2007; Carlquist 2009). The sympodiality and laxity observed in the stems of these cane-like shrubs can be directly assessed by the analysis of their architectural and mechanical properties. Stem mechanical properties can be used to characterize different growth forms based on the observation of mechanical shifts in structural Young's modulus (*E*) and flexural rigidity (*EI*) during development (Rowe and Speck 2005; Lahaye *et al.* 2005). *Amborella* differs from most of the other ‘basal’ cane-like angiosperm representatives in that it has a vesselless wood, containing only tracheids as water-conducting cells (Feild *et al.* 2000). It has been argued that vessel evolution represents a major leap in angiosperm diversification by increasing developmental options for hydraulic, mechanical and storage functions (Sperry *et al.* 2007; Isnard and Feild 2015). Therefore, the analysis of the stem mechanical properties of *Amborella* can be potentially important in understanding the mechanical organization behind the scandent habit in vesselless plants.

Independent of the multiple forms expressed by plants, several leading dimensions of trait covariation have been documented (Ackerly and Donoghue 1998; Enquist 2002; Niklas and Enquist 2002; Westoby *et al.* 2002; Wright *et al.* 2004; Olson *et al.* 2009; Diaz *et al.* 2016). These apparently highly homoplasious patterns of trait variation appear to span most flowering plant lineages given that they are observed across species and across habitats. One of the best documented of these relationships is the ‘leaf size-twig size’ spectrum (Ackerly and Donoghue 1998; Cornelissen 1999; Westoby *et al.* 2002; Westoby and Wright 2003; Sun *et al.* 2006; Wright *et al.* 2007; Olson *et al.* 2009), which includes ‘Corner's Rules’ (Corner 1949). The leaf size-twig size spectrum includes the tendency for plants with large leaves to have predictably thick twigs made up of tissues with low specific density (Wright *et al.* 2006; Swenson and Enquist 2008; Olson *et al.* 2009). Finding whether *Amborella* fits into these global patterns helps understand how widespread these patterns are across the angiosperms. Although these relationships are predictable, the absolute values of functional traits can vary across species, generating different ecological strategies. Within the context of these strategies, phenotypic plasticity involves modification of developmental trajectories in response to environmental cues (Sultan 2000; Chambel *et al.* 2005; Pigliucci *et al.* 2006; Fusco and Minelli 2010).

Among the environmental variables that influence plant phenotypic plasticity, light availability is one of the most heterogeneous (Valladares and Niinemets 2008). Light incidence has a very well documented influence on leaf structure. For instance, light tends to have a negative effect on leaf size (Poorter 1999; Rozendaal *et al.* 2006) and a positive effect on specific mass (Abrams and Kubiske 1990). Other changes induced by light availability include mass allocation (Poorter *et al.* 2012) and overall plant architecture (Kawamura and Takeda 2002; Charles-Dominique *et al.* 2010; 2012). Given that selection seems to favour thicker twigs as leaf size increases, and because light has a documented effect on leaf size, then we can expect that light indirectly influences stem size. Hence, plants should be able to respond plastically to differing light environments along a given stem-leaf scaling slope, moving to different degrees along the leaf size-twig size spectrum. Along with the conspicuous effect of light variability on foliar traits, different light regimes can also induce changes in stem structure. It has been shown that shade conditions induce the elongation of internodes and petioles as part of a common shade avoidance response (Schmitt *et al.* 2003; Huber *et al.* 2014). Shade-induced elongation seems to be coupled with other structural changes. For instance, it has been suggested that plants growing under shaded conditions tend to have a higher modulus of elasticity (MOE), producing more rigid stem tissues (Gallenmüller *et al.* 2004; Anten *et al.* 2005; Watari *et al.* 2014; Huber *et al.* 2014).

A previous study has shown that *Amborella* individuals growing in different light environments exhibit variations in leaf thickness and orientation (Feild *et al.* 2001). Nevertheless, Feild *et al.* (2001) reported an absence of variation in leaf area (LA)-specific hydraulic conductivity and in photosynthetic light use, concluding that *Amborella* has limited developmental flexibility in response to light flux density variation. However, no study to date has examined possible plastic responses of *Amborella* in architectural and mechanical organizations, as well as in ecologically informative functional traits such as leaf mass per area (LMA), leaf dry matter content (LDMC), stem specific density (SSD), and stem water content (SWC). Analyzing the influence of light on functional traits, and the coordination between these traits, can help us to understand to what degree *Amborella* is able to respond to light variability. To the extent that structural variation in *Amborella* fits into the currently known spectra of variation across the flowering plants, then this would increase our confidence that the potential for plastic variation along these axes was present in the angiosperm ancestor.

The current study addressed the overall architecture and mechanical behaviour along with 12 leaf and stem functional traits of *A. trichopoda*. Sampling was carried out on individuals growing under different canopy opennesses to assess the effects of light variability on functional trait and morphological plasticity. We used our observations on *Amborella* to estimate the pervasiveness of leaf and stem economics and trait scaling, which seem to be widespread across the angiosperms. Finally, we discuss our results in the context of angiosperm growth form evolution.

## Methods

### Plant material, study sites and sampling

*A.* *trichopoda* is a woody evergreen shrub 6- to 9-m tall, which grows in the understory of the rainforest of the central mountain range of New Caledonia on acidic substrates at 100- to 900-m elevation (Jérémie, 1982). *Amborella* is dioecious with small (3–5 mm) flowers that are wind/insect pollinated, and it grows in small, male-biased populations with measured densities of 433 individuals per ha (Thien *et al.*, 2003). Our architectural observations were carried out on individuals from a population in the natural reserve of Mount Aoupinié in the Northern province of New Caledonia. Mt. Aoupinié has one of the populations of *Amborella* with the highest levels of genetic diversity (Poncet *et al.* 2013). A forestry road runs east to west along the ridge of Mt. Aoupinié, and the associated clearing has exposed several individuals to a considerable increase in light conditions compared to nearby forest populations. Seedlings and young individuals growing in the greenhouses of the *Institut Agronomique Néo-Calédonien* (IAC) at Saint Louis, Mont Dore, New Caledonia, were also used for architectural observations.

To evaluate stem and leaf economics within *Amborella*, we measured nine stem and three leaf structural and functional variables (Table 1). Outer canopy branches were collected along a gradient from sun-exposed roadside individuals to individuals growing in the shaded forest understory. We sampled 24 peripheral branches bearing all of their distal leaves for allometric analysis and for stem and leaf trait measurements. We selected branches bearing fully expanded leaves, avoiding leaves with pathogens or herbivore damage. Additional segments were collected for mechanical and stem trait analyses. To avoid desiccation, sampled stems were collected predawn. Stems were immediately defoliated and wrapped in moist paper towels, sealed in plastic bags and stored in the dark at 7 °C for transport.

**Table 1.** Structural and functional stem and leaf traits measured.

Trait	Abbreviation	Units
Stem length	SL	cm
Internode length	IL	cm
Internode diameter	ID	cm
Length-diameter ratio	LDR	–
Number of leaves	NL	–
Leaf area	LA	cm^2^
Leaf mass per area	LMA	g m^−2^
Leaf dry matter content	LDMC	mg g^−1^
Stem specific density	SSD	g cm^−3^
Stem water content	SWC	%
Modulus of elasticity	MOE	N mm^−2^
Modulus of rupture	MOR	N mm^−2^

### Measurement of canopy openness

Canopy openness (CO, in %), which represents the percentage of open sky at a given point, is a useful index of the light environment experienced by a given plant (Jennings *et al.* 1999). Canopy openness was measured to assess the effect of light availability on the structural and functional properties of *Amborella*. We used hemispherical photographs to characterize local CO at each sampled branch. Before collecting each branch, three photographs were taken above the basal, medial and apical branch section using a 180° hemispherical lens (Samyang fisheye 8 mm f/3,5. Samyang, South Korea) mounted on a Canon EOS 7D camera body (Canon, Japan). The reported CO for a given branch is the average of the three photographs. Photographs were taken between 11 and 13 h, preferentially on cloudy days. The resulting images were analyzed using gap light analyzer software (Frazer *et al.* 1999).

### Architectural analysis

Plant axes were described morphologically and illustrated following the criteria of Barthélémy and Caraglio (2007) and Charles-Dominique *et al.* (2010, 2015). The architectural description focused mainly on aboveground structure. Axes were categorized in terms of (i) growth process (whether axes have an indeterminate (monopodial) or a determinate (sympodial) growth pattern), (ii) growth direction (whether axes have an erect (orthotropic) or an horizontal (plagiotropic) general orientation), (iii) branching pattern (whether branches elongate immediately after bud initiation or originate from dormant buds with a delayed extension), (iv) branch position (whether branches are located at a basal (basitonic), medial (mesotonic) or distal (acrotonic) position on the parent axis) and (v) symmetry (whether the leaves and branches are disposed radially or bilaterally). Below, we use the term ‘module’ (*sensu* Charles-Dominique *et al.* 2015) to denote a structural unit repeated over time and made up of a single dominant axis and its lateral subordinates axes. Our architectural analysis was based on *in situ* observations of individuals at different growth stages, defined *a priori* on the basis of morphological criteria (Charles-Dominique *et al.* 2010). Some of these criteria included branching and accumulation of relays. ‘Relay’ is used here to denote axes that originate from dormant buds and that grow into new sets of modules. Relays accumulate over time, providing a basis for classifying individuals into different stages (Charles-Dominique *et al.* 2010). Regardless of the environment in which they are growing, individuals from older stages have more relays than those of earlier stages. Age of individuals was estimated by comparison with field and greenhouse individuals of known age. Through architectural and morphological descriptors, we described differences between mature individuals growing in various light environments.

### Leaf traits and branch dimensions

We measured LA, LMA and LDMC of all the leaves, petioles included, borne by the 24 branches sampled. This sampling allowed us to determine the total LA for each branch. Leaves were scanned in the field using a portable scanner (CanoScan LiDE 25, Canon, Japan), and fresh mass was immediately measured using an analytical balance. Leaf area was calculated from the scanned images using ImageJ 1.47v. (NIH Image, Bethesda, MD, USA). Leaves were then oven dried at 70 °C for 72 h, for LMA and LDMC calculations. Leaf mass per area was calculated as the ratio of leaf dry mass to LA; LDMC was calculated as leaf dry mass over leaf fresh mass (Pérez-Harguindeguy *et al.* 2013). Branch measurements included number of leaves, total stem length (SL), internode length (IL), internode diameter (ID), and the ratio of SL to stem diameter, which was calculated as SL over ID of the basal-most internode. Internode length and ID measurements were made at each internode of the sampled branches.

### Stem mechanics

We measured MOE, also known as Young's modulus, along with modulus of rupture (MOR), and flexural rigidity (*EI*) of stem segments from the same branches sampled for the measurements of leaf traits. To cover the widest possible range of stem thickness given our testing apparatus, we sampled additional stems of wider diameters, which were included in a separate dataset. We measured a total of 100 stem segments with diameters ranging from 1.97 to 22 mm. Segments were tested in three-point bending with an Instron InSpec 2200 test machine fitted with 10-, 125- or 500-kN load cells (Instron Corporation, Norwood, MA, USA). Stem segments had length: diameter ratios of 20:1 to avoid shear (Lahaye *et al.* 2005; Méndez-Alonzo *et al.* 2012). The diameter of the tested segments was calculated as the average of the basal, midpoint and apical diameters measured at two perpendicular points with a digital caliper. The axial second moment of area (*I*) was calculated assuming that the stem cross section is approximated as a solid ellipse by Equation (1):

(1) $$I\;=\left(\frac{\pi }{4}\right)\cdot \left(r_{1}^{3}\cdot r_{2}\right)$$

where *r_1_* is the radius of the stem in the direction of the applied force and *r*_2_ is the radius in the perpendicular direction. Stem flexural rigidity (*EI*) represents the resistance of a beam to bending forces in terms of size, geometry, and material properties. It was calculated using Equation (2):

(2) $$EI=\frac{L^{3}}{48m}$$

where *L* is the distance between the supports of the testing apparatus and *m* is the slope of the initial elastic portion of the deflection vs. force curve. For MOE and *EI* calculation, a force was applied at a speed of 0.25 mm/s, inducing a displacement of 2.5 mm. Modulus of elasticity is an index of the capacity of a material to resist bending assuming that the stem is made up of a uniform material. Modulus of elasticity was calculated with Equation (3):

(3) $$MOE=\frac{EI}{I}$$

MOR, also known as flexural strength, represents the highest stress experienced by the stem at its moment of rupture. In MOR tests, load displacement was conducted until reaching maximal force (*F*_max_, the maximum load at the moment of breakage or the limit of the elastic phase in absence of breakage). *F*_max_ was calculated with software IX Instron System (Instron Corporation, Norwood, MA, USA). *F*_max_ was used to calculate MOR using Equation (4):

(4) $$MOR=\frac{\left(F_{max}\;\times L\;\times r\right)}{4I}$$

where *L* is the length between the supports, *r* is the radius and *I* is the second moment of area (Gere and Timoshenko 1999; Méndez-Alonzo *et al.* 2012).

### Stem density and SWC

We collected stem samples 2.5- to 3-cm long from central sections of the segments tested mechanically. Stem volume was calculated using the water displacement method. We oven-dried stem samples at 70 °C for a minimum of 72 h until mass was constant. Stem specific density (SSD) was calculated as dry mass/fresh volume. Stem water content was calculated following Poorter *et al.* (2010) as 100 (1 − (dry mass/fresh mass)). To test the hypothesis that branches with greater LA have stems with lower tissue density, we used an approach similar to that of Wright *et al.* (2006) by measuring SSD of the apical-most branch sections, taking exclusively stem segments collected < 350 mm from the branch tip. Apical stem density should reflect the conditions prevailing during the production of the standing crop of leaves, and therefore should correlate well with leaf characteristics.

### Data analysis

All analyses were conducted in R v.3.1.2 (http://www.r-project.org). Functional and structural variables were log-transformed to meet assumptions of normality and homoscedasticity. Two datasets were assembled. The first, hereafter referred to as the ‘branches’ dataset, contained arithmetically averaged values of all of the traits measured (Table 1) for each of the sampled branches. The second dataset, hereafter referred to as the ‘biomechanics’ dataset, contained values for the stem mechanical traits along with SSD, SWC and CO values of 100 measured segments. The ‘biomechanics’ dataset was used to analyze the variation in mechanical properties on a wider range of stems diameters, allowing us to test predictions regarding mechanical variation during development.

Given that we observed architectural differences under different CO, especially in terminal sympodial modules, we explored whether there were changes in scaling between stem dimensions and total LA between light environments. We divided our dataset into two different light environment sites taking 15 % CO as a threshold, using a ‘sun/shade’ site categorical variable. This CO threshold was chosen because all of the branches collected in the understory (shade site) had values of *<*15 % CO while branches sampled in forest clearings along the road (sun site) had values of  >15 % CO. Allometric scaling between stem size (diameter and length) and total LA under different CO values was estimated in log-log bivariate relationships using standardized major axis (SMA) regressions using the R package 'smatr' (Warton *et al.* 2012). We built a model predicting total LA based on stem diameter, site, and a stem diameter–site interaction term. A second model predicted total LA based on SL, site, and a SL–site interaction term. Using these models we estimated the relationship between stem size and total LA across CO sites, and we compared scaling slopes of sun branches with those of shade branches via likelihood ratio statistics for common slopes. After finding that there were no slope differences between sites, we fit models without the site interaction term. We then used Wald statistics for equal intercepts included in the 'smatr' package to compare intercepts between sites. Similar slopes but different intercepts indicate that stem size differs significantly between sites but foliage-stem scaling is similar. Standardized major axis regression was also used to assess the relation between apical SSD and LA. Standardized major axis is designed to describe relationships between variables in which the causality of one on the other is likely mutual rather than unidirectional one variable on the other making it appropriate for the present situation (Smith 2009).

We explored relationships between traits using pairwise Pearson correlations on the ‘branches’ and ‘biomechanics’ datasets. Separately, the effect of CO on the modulation of leaf traits was analyzed with ordinary least squares linear regressions (OLS) on the ‘branches’ dataset. To document changes in stem material mechanical properties with ontogeny, we measured the effect of stem diameter on MOE and MOR using OLS regressions on the ‘biomechanics’ dataset. To explore a possible joint effect of stem diameter, SSD and CO on stem mechanical properties, we performed multiple regression analyses on the ‘biomechanics’ dataset. The strength of the contribution of each stem trait and of CO was evaluated using semipartial correlations.

## Results

### Architectural analysis

**Figure 1.**
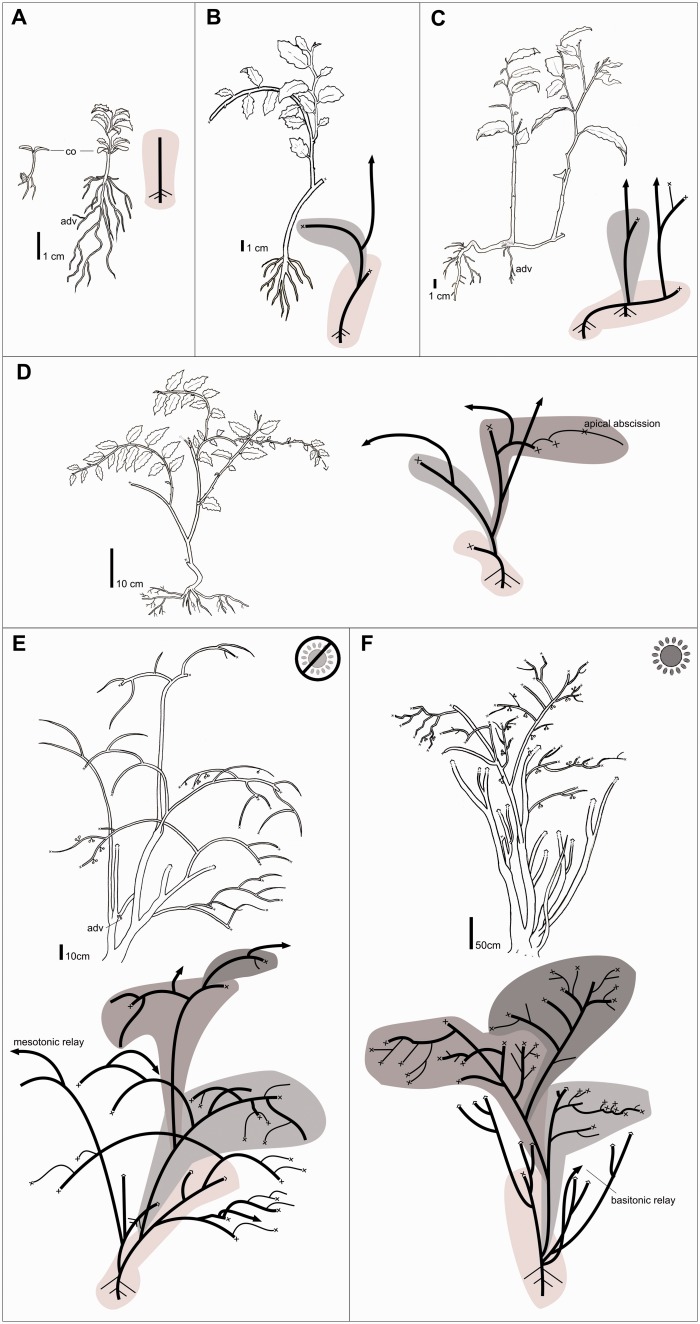
Illustration of ontogenetic architectural stages of *Amborella trichopoda*, and architectural variability under closed or open canopies. (A) Seedling and unbranched young plant 6 months after germination (stage 1). (B) 1-year-old plant (stage 2). (C) 1.5-year-old plant with a rooted ‘pseudo-rhizome’ (stage 2). (D) Around 6-year-old plant (stage 3). (E) >10-year-old plant growing under a closed canopy (stage 4). (F) >10 year-old-plant growing under open canopy (stage 4). Only one sequence of the successive modules of stage 4 individuals is represented. Abbreviations: adv, adventitious root; co, collar zone. Thick lines represent structural axes, thin lines represent lateral branches, arrowhead lines represent delayed relays, crosses are dead apices, circles are inflorescences, and gray shadings indicate successive architectural modules.

***Stage 1–small seedlings***. *Amborella* seedlings have a tap root and a single orthotropic stem with alternate spiral phyllotaxy (Fig. 1A). After about 12 weeks, as observed in the greenhouse, seedlings have a stem 5-cm tall and a large root system (Fig. 1A).

**Figure 2:**
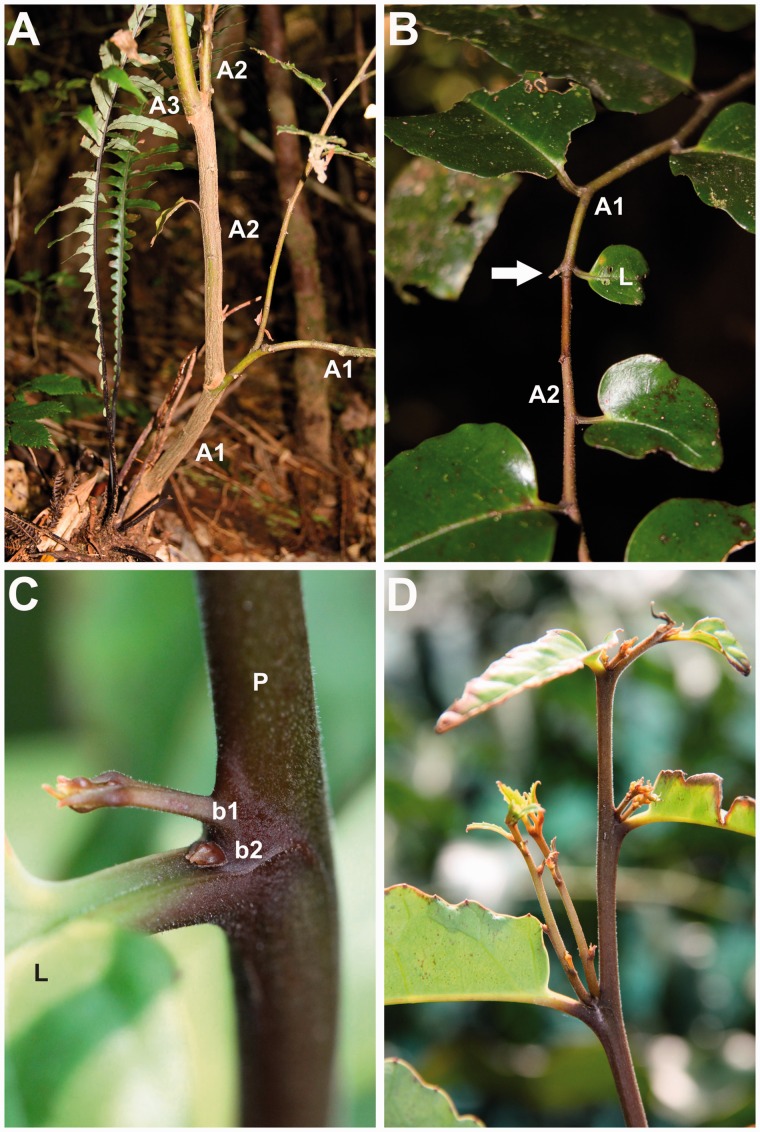
Illustration of morphological features of *Amborella trichopoda*. (A) Young individual growing in the understory showing sympodiality by stacking of modules. Note the changes in diameter of the parent axes (A1, A2) after the branching. (B) Linear sympodial structure after apical death (arrow). A second axis produced by the lateral bud of the more distal leaf continues axis construction keeping the same growth direction. (C) A supernumerary bud is located below an axillary bud showing onset of growth. (D) Under high-light conditions, both axillary buds can activate, producing small axes. Abbreviations: A1, A2, A3, axis orders; b1, axillary bud; b2, supernumerary bud; L, axillary leaf; P, parent axis.

***Stage 2–young saplings***. As the first orthotropic axis elongates, it becomes plagiotropic (i.e. becomes a ‘mixed’ axis, with both orthotropic and plagiotropic sections) becoming pendulous under its own weight (Fig. 1B). Phyllotaxy is alternate, oriented spirally in the proximal orthotropic section. Leaf orientation is bilaterally symmetrical in the distal plagiotropic section. A lateral mixed axis makes up a second architectural module. Branching is sympodial. In most of the individuals we observed a single branch developed after apical death of the parent axis (Fig. 1B and C). Apical death occurs mainly after the bending of axes (Fig. 1B). Branching is predominantly mesotonic and on the upper surface of the bending zone (Fig. 1B). The basal diameter of the lateral branch becomes equivalent to that of the section of the parent axis preceding the branching (Fig. 2A). As the successive module develops, the distal part of the parent axis withers and decays (Fig. 1B and C). Relay branches sprout on the second module originating from dormant mesotonic buds (Fig. 1B and C). In some individuals, the first module comes to lie on the ground. The lying stem can develop adventitious roots and resprout and then becoming a ‘pseudo-rhizome’ from which several stems develop (Fig. 1C).

***Stage 3–early maturity***. Larger individuals grow continuously with a sympodial branching pattern (Fig. 1D). Only some nodes produce lateral branches, and there is no obvious regular distribution of branches in tiers (Fig. 1D). Branch production seems to be associated with local environmental conditions, given that we observed increases in lateral branch production in modules growing under light patches and after trauma such as falling branches. All axes are morphologically similar and we did not observe a hierarchical architectural construction with distinctive axis categories (Fig. 1D), unlike as in conifers, which have distinct central and lateral stems. Axes derived from the relay stem originating from the second module establish a successive sympodial module (Fig. 1D). At this stage, acrotonic sympodial branching can occur in peripheral branches after apical abscission of the parent axis (Figs. 1D and 2B). The axillary bud of the terminal leaf activates, producing a new axis that maintains the same growth direction (Fig. 2B).

***Stage 4–maturity***. Individuals at this stage are built by the sequence of over four sympodial modules (Fig. 1E and F). Modules are formed by a combination of branch-bearing stems and leaf-bearing lateral branches. This sequence of modules is reiterated by mesotonic and basitonic relays originating from dormant buds. As a consequence of the accumulation of iterated complexes of sympodial modules, the plant has a multi-stemmed shrub form and a leader stem is not distinguishable (Fig. 1E and F). Flowering is lateral, occurring on both stems and lateral branches. No architectural differences were observed between male and female individuals. Adventitious roots were frequently observed above the ground at the stem base (Fig. 1E).

***Morphological differences across canopy opennesses*.** Given a lack of recruitment in sun-exposed sites, we observed stages 1 to 3 only in shaded understory conditions. We observed qualitative morphological variation in modules under different canopy opennesses in large (stage 4) individuals. Modules of plants growing in more shaded environments were made up of very elongate and sparsely branched axes (Fig. 1E), whereas modules in open canopy environments had more lateral branches (Fig. 1F). Under closed canopy, several relays occurred mostly at mesotonic positions, whereas under open canopy relays were less frequent and were usually basitonic. Under open canopy conditions, axillary supernumerary meristems activate (Fig. 2C). Supernumerary axillary buds produce additional short branches (Fig. 2D). The lifetime of these short axes seems to be very short, given that we observed a frequent abscission of small branches in terminal modules of individuals growing under open canopies (Fig. 1F).

### Foliage-stem scaling in *Amborella*

**Figure 3:**
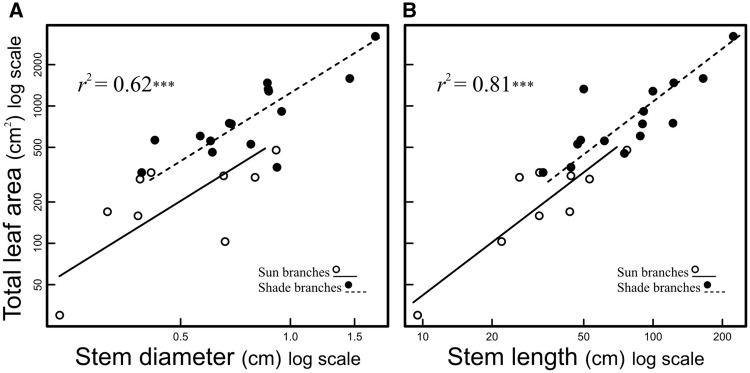
SMA regressions showing the scaling of total leaf area (sum of areas of all leaves borne by the stem) with stem dimensions of branches growing under open canopy (>15 % CO) and closed canopy (<15 % CO). (A) Total leaf area and stem diameter allometry; (B) total leaf area and SL allometry. ***P < 0.001.

Both stem diameter and length were significantly related to total LA (Fig. 3). Stem diameter, equivalent to the diameter of the basal-most and thickest internode of each branch, predicted 62 % of the variation in total LA across light environments (Fig. 3A). Stem length was also strongly related to total LA, explaining 81 % of its variation (Fig. 3B). When assessing differences in the scaling of stem diameter and total LA among sites, the model indicated that both types of sites have similar SMA slopes (likelihood ratio (1) = 0.16; *P* = 0.68). Standardized major axis slopes ranged from 1.58 (95 % confidence intervals (CIs) 1.10–2.27) for shade branches to 1.82 (95 % CIs 0.97–3.41) for sun branches. Assuming homogeneity of slopes, branches from shade exposed sites had an intercept of 3.08 (95 % CIs 2.96–3.21), which was not quite significantly higher (Wald's test (1) = 5.51; *P * = 0.01) than the intercept of sun-exposed branches (2.85; 95 % CIs 2.39–3.32). Biologically, this result indicates that similar allometric scaling relationships are maintained regardless of the light environment, with sun branches having slightly thinner stems. With regard to SL, SMA slopes of sun branches (1.39; 95 % CIs 0.92–2.10) and shade branches (1.17; 95 % CIs 0.83–1.66) were not significantly different (likelihood ratio (1) = 0.47; *P *= 0.48). Intercepts were also similar (Wald's test (1) = 1.77; *P*  = 0.18) between sun branches (0.16; 95 % CIs −0.74 to 0.92) and shade branches (0.66; 95 % CIs −0.12 to 1.46). In addition to the LA and stem size scaling, total leaf number was also significantly correlated with SL and diameter (Table 2).

**Table 2.** Pairwise Pearson correlations between stem and leaf traits and canopy openness.

	SL	IL	ID	LDR	NL	LA	LMA	LDMC	SSD	SWC	MOE	MOR
IL	0.85***											
ID	0.74***	0.77***										
LDR	0.74***	0.58***	0.23									
NL	0.86***	0.55**	0.53**	0.62***								
LA	0.77***	0.89***	0.73***	0.38	0.57***							
LMA	−0.49**	−0.60***	−0.19	−0.46*	−0.29	−0.62***						
LDMC	−0.39	−0.59***	−0.25	−0.34	−0.14	−0.56***	0.89***					
SSD	−0.25	−0.36	−0.11	−0.26	−0.06	−0.33	0.48*	0.53*				
SWC	0.26	0.35	0.10	0.25	0.09	0.39	−0.67***	−0.69***	−0.85***			
MOE	0.66**	0.59***	0.67***	0.36	0.54**	0.59**	−0.12	−0.01	0.46***	−0.49***		
MOR	0.51*	0.44*	0.34***	0.34	0.55**	0.48*	−0.07	0.03	0.56***	−0.59***	0.72***	
CO	−0.49*	−0.73***	−0.31	−0.35	−0.21	−0.72***	0.88***	0.85***	0.35	−0.53**	−0.21	−0.06

Correlations based on averaged values of 24 sampled axes. See Table 1 for traits abbreviations and units. Correlations between MOE, MOR, ID, SWC and SSD were calculated using the ‘biomechanics’ dataset (*n* = 100). Significant correlations are shown in bold.

* *P* < 0.05;

** *P* < 0.01;

*** *P* < 0.001.

### Coordination of leaf and stem functional traits, and effect of canopy openness

**Figure 4:**
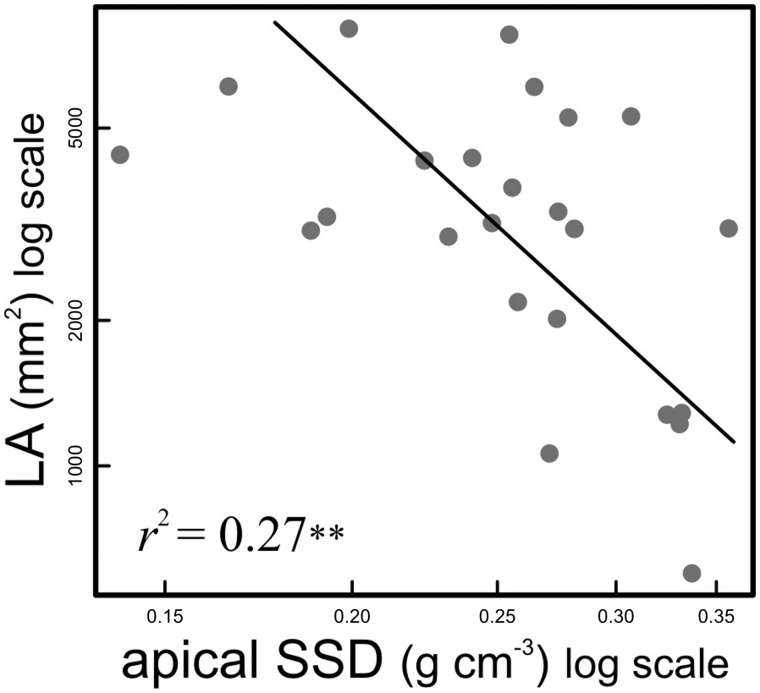
SMA regression showing the relationship between SSD of apical stem sections and mean LA of the leaves subtended by each stem (**P = 0.008; slope −2.83, 95 % CI − 4.09 to − 1.96).

**Figure 5:**
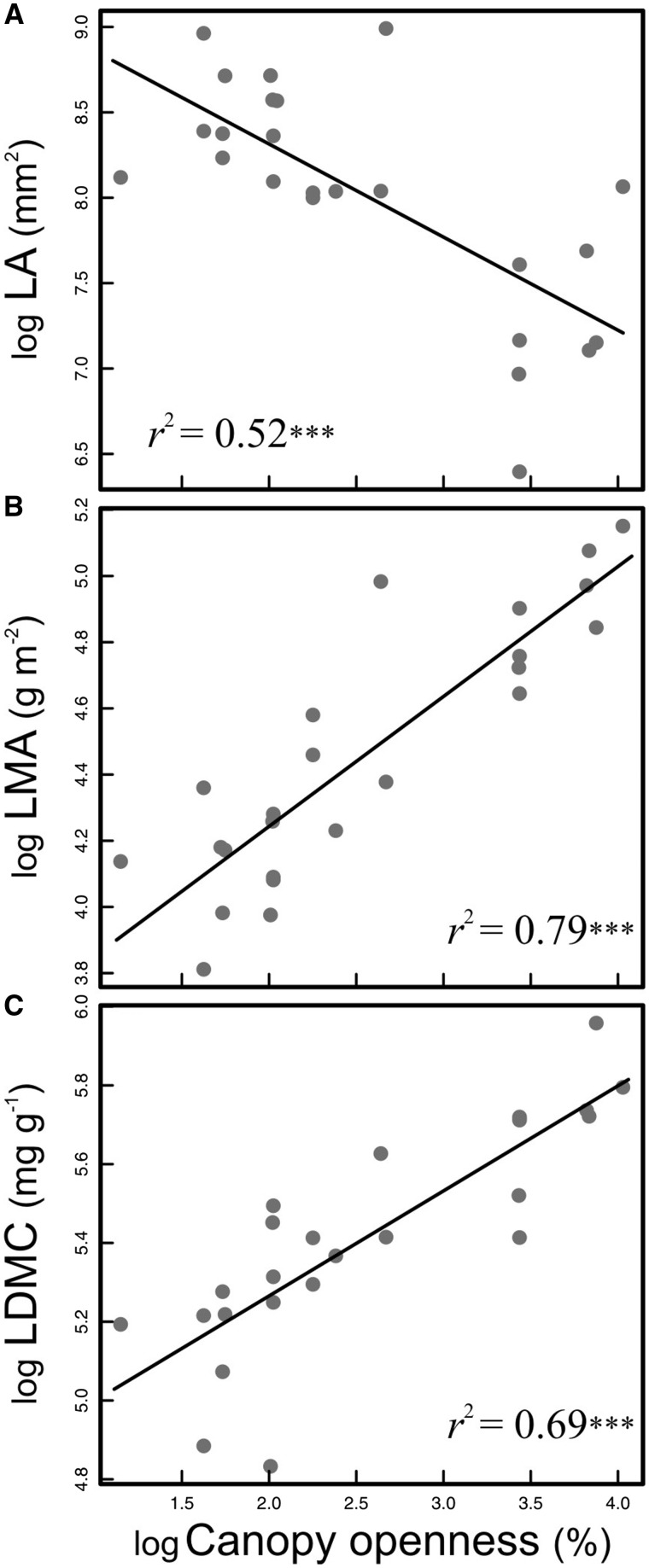
Influence of canopy openness on leaf trait variation. (A) Decrease in leaf area with increasing canopy openness. (B) Increase in leaf mass per area with increasing canopy openness. (C) Increase of leaf dry matter content with increasing canopy openness. *n* = 24. ***P < 0.001.

Most stem and leaf traits were significantly correlated (Table 2). Leaf dry matter content and LMA had the strongest relationships with stem traits such as SWC and SSD. Leaf area was strongly correlated with the remaining leaf traits, as well as with most of the stem traits measured (Table 2). Stem size traits such as SL, IL and ID were very strongly correlated with leaf traits and stem mechanical traits, but were not correlated with SSD and SWC. Stem specific density was positively correlated with both MOE and MOR. Stem water content and stem mechanical properties were negatively correlated, indicating that stems with higher water contents had tissues that were both more flexible and less resistant to breakage. Branch averaged values of SSD and LA were not significantly correlated (Table 2). However, average LA was negatively correlated with SSD of branch apical segments (Pearson *r* (22)* * = −0.52, *P*  = 0.008), and scaling between both traits was detected (Fig. 4), indicating that broad leaved branches of *Amborella* have stem tissues of lower density. Canopy openness, which reflects light availability, was significantly correlated with leaf and stem size traits (Table 2). Stem water content was negatively associated with CO (Table 2), suggesting lower water contents in sun-exposed branches. Canopy openness had a very important effect on leaf trait variation (Fig. 5). Leaf area was negatively related to CO (Fig. 5A), whereas CO was strongly positively related to both LMA (Fig. 5B) and LDMC (Fig. 5C). Sun-exposed leaves were smaller than shade leaves, but had higher mass per unit of area and higher dry matter content than leaves under closed canopy.

### Stem mechanics

**Figure 6:**
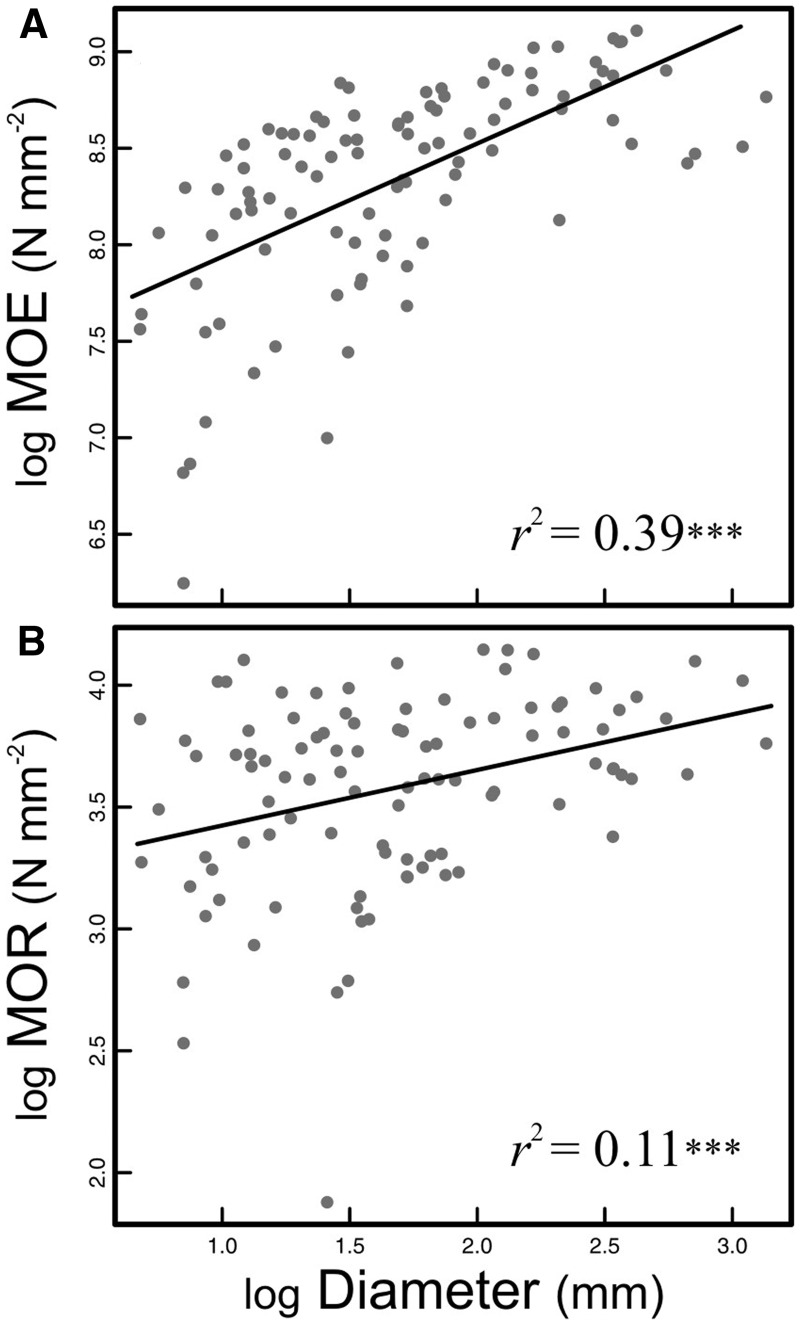
Relationships between mechanical properties and stem diameter. (A) Increase in stem stiffness (MOE) with increasing diameter. (B) Increase in stem resistance to breakage (MOR) with increasing diameter. *n* = 100. ***P < 0.001.

Mechanical parameters (MOE and MOR) were significantly predicted by stem diameter (Fig. 6). Modulus of elasticity increased with stem diameter (Fig. 6A) from 500 to 2000 N mm^−^^2^ in stems of 1.98–2.5 mm to 7000–9000 N mm^−2^ in stems with diameters of  >10 mm. Because higher values of MOE reflect higher material stiffness, this result indicates that tissues in thicker basal stems are stiffer than those in narrow apical ones. As regards MOR, narrower stems had lower resistance to rupture whereas wider stems were more resistant to rupture (Fig. 6B). Flexural rigidity (*EI*) of *Amborella* stems was strongly correlated with diameter (*r*^2^ (98)  = 0.97; *P* < 0.001), indicating that higher loads were needed to produce deflection in stems of largest diameters.

Multiple regression analysis on the biomechanics dataset including the effect of stem diameter, SSD and CO (Table 3) on stem mechanics showed a significant variation of stem mechanical parameters with CO. However, when explaining MOR, the coefficient associated with CO was not significant (Table 3). As for MOE and *EI*, the negative coefficient associated with CO was significant suggesting a decrease of stem tissue rigidity under open canopy. Despite the significance of these coefficients, CO was the variable that contributed least to the models, as shown by the lower semipartial correlation values when compared to those of the other parameters (Table 3). Modulus of elasticity and *EI* increased with both stem diameter and SSD, with shade axes tending to be stiffer. With regard to MOR, SSD had the highest semipartial correlation, explaining more of the total variation in MOR than stem diameter (Table 3). The lower association of MOR with stem diameter, as compared to the association between MOE and diameter, was readily observed in the scatter plots (Fig. 6). Stem specific density also had a significant effect on MOE, but it had a lower semipartial correlation than stem diameter (Table 3). Suggesting that SSD plays a major role in stem resistance to rupture and a lesser but still important effect on stem flexibility.

**Table 3.** Multiple regressions of *Amborella* mechanical properties predicted by canopy openness (CO), stem diameter (D), and stem specific density (SSD).

Response	*r*^2^	*F*test	βD	βSSD	βCO	PD	PSSD	PCO
MOE	0.55	*F*_3,96_ = 39.56***	0.47***	1.00***	−0.18**	0.47	0.38	0.22
MOR	0.41	*F*_3,96_ = 22.51***	0.12*	1.03***	−0.08^ns^	0.18	0.54	0.13
*EI*	0.98	*F*_3,96_ = 1717***	4.46***	1.00***	−0.18**	0.91	0.08	0.04

*r*^2 ^= adjusted coefficient of multiple determination. βD =  coefficient associated with stem diameter; βSSD = coefficient associated with stem specific density; βCO = coefficient associated with canopy openness. PD, PSSD, and PCO are semipartial correlations indicating the contribution of each predictor (*n* = 100). ns = non significant;

* *P* < 0.05;

** *P* < 0.01;

*** *P* < 0.001.

## Discussion

*A.* *trichopoda*, the sister species to all other flowering plants, should not be regarded as an archetype of the angiosperm ancestor simply because of its phylogenetic position (Crisp and Cook 2005). However, our observations of the structural and functional variation of *Amborella* can provide insights into the patterns of distribution of characters along major clades. *Amborella* varies predictably in functional and structural traits with light environment. Within this variation, leaf and stem economics are coordinated. For example, though the axes of *Amborella* varied in LMA and LDMC under variation in canopy openness, they maintained similar foliage-stem scaling. The confirmation of these patterns of covariation in *Amborella*, together with their wide distribution across both the angiosperms and conifers, suggests that these coordinated plastic responses were likely part of the basic developmental toolkit in the ancestral angiosperm. Here, we discuss some of the patterns of trait coordination that are widespread in plants in the context of the *Amborella* growth form, and what these characteristics might indicate regarding angiosperm synapomorphies or symplesiomorphies.

### Corner's rules and *Amborella* growth form plasticity

Plants vary from species with thick twigs bearing large leaves to species with narrow twigs bearing small leaves (Westoby and Wright 2003). Here, we show that this spectrum can be observed at the intraspecific level in *Amborella*, which exhibit similar foliage-stem scaling across light environments (Fig. 3). It has been proposed that foliage-stem scaling is a consequence of the mechanical and hydraulic requirements of leaves, as well as self-shading avoidance through leaf spacing (Enquist 2002; Westoby *et al.* 2002). Moreover, if similar crown areas fix similar amounts of carbon, then carbon limitation requires such a foliage-stem scaling if leaf spacing is greater in larger-leaved species (Olson *et al.*, 2009). In addition to foliage-stem scaling in *Amborella*, we observed an association of low SSD with stem elongation, high LA, and leaf spacing. Across light environments *Amborella* has narrow stems of high density bearing small leaves with high mass per area or thick branches of low densities bearing wide leaves with low mass per unit area. Our results thus converge with the metabolic mechanism proposed by Olson *et al.* (2009), suggesting that if leaves and stems maintain a metabolically driven proportionality, large-leaved axes with greater leaf spacing require low density tissues and thicker stems, as a response of rapid volumetric extension and stem tissue mechanics given carbon limitation.

A second component of Corner's rules implies that species with larger leaves and twigs also tend to have less frequent branching with wider branching angles, whereas species with smaller leaves and twigs have more frequent branching with narrower branching angles (Corner 1949; Ackerly and Donoghue 1998; Westoby and Wright 2003). Our architectural analysis shows that the crowns of *Amborella* individuals have conspicuous morphological differences depending on light environment. Crowns of individuals growing under closed canopy have sparser branching and few, long lateral branches. Crowns of individuals growing under open canopy show an increase in branching frequency. The greater branching of *Amborella* individuals growing under high-light conditions is promoted by the activation of supernumerary buds leading to a short, densely leaved, and narrow crown. Similar crown morphological responses to light availability have been observed across shade-tolerant angiosperm species (Cornelissen 1993; Niinemets 1996; Kawamura and Takeda 2002). The observed variation in the architecture of *Amborella* individuals under different canopy opennesses suggests that intraspecific architectural plasticity follows Corner's Rules.

### Phenotypic plasticity of *Amborella* leaf and stem traits in response to canopy openness

Numerous studies spanning a wide diversity of plant lineages have highlighted that leaf characteristics can be strongly influenced by local light environment. Here, we extend this documentation to *Amborella*, whose leaves vary markedly in size and mass allocation under different levels of canopy openness (Fig. 5). This variation in leaf traits in different light environments is very likely adaptive. For instance, *Amborella* leaves in shade conditions have greater area for a given unit of biomass, likely increasing the surface available for light interception (Poorter *et al.* 2009). Lower LMA in understory *Amborella* leaves reflects a reduction in the cost of leaf construction. It has been shown that lower construction investment is favoured in environments with low photosynthetically active radiation (Poorter *et al.* 2006). In addition to light availability, canopy openness is also positively related to air temperature, and negatively related to relative humidity (Pineda-García *et al.* 2013). Smaller leaves have a smaller boundary layer, which allows for more rapid convective heat loss (Parkhurst and Loucks 1972). As a consequence, smaller leaves might lower transpiration and water loss. Lower LA, along with the increases in LDMC and LMA that we observe in *Amborella*, can be therefore considered as adaptive responses to potential overheating and desiccation under open canopy conditions (Niinemets *et al.*, 1999).

Several mechanisms could underlie the variation we observed in the absolute values of LMA in response to light availability (Fig. 5B). It has been proposed that leaf tissue density is strongly correlated with LMA in woody plants (Castro-Díez *et al.* 2000; Villar *et al.* 2013), and leaf tissue density seems to predict LMA better than leaf thickness (Villar *et al.*, 2013). A previous study suggested that *Amborella* leaf epidermal and hypodermal thickness do not change in response to varying light levels (Feild *et al.* 2001). Feild *et al.* (2001) also reported limited adjustments in total leaf thickness, with sun leaves being *ca*. 10 % thicker than shade leaves. Our results, however, show a very strong effect of light availability on LA and mass investment (LMA and LDMC) (Fig. 5B and C). The variability we observe in LMA and LDMC suggests that if *Amborella* leaf thickness is not highly variable, as suggested by Feild *et al.* (2001), light incidence may induce significant shifts in tissue density. Further studies would be needed to detect potential structural changes at the cellular level in both the epidermis and the mesophyll of leaves. This could provide information on the leaf constituents that drive *Amborella* LMA variation under different light environments.

The plastic responses of leaf size and leaf mass allocation to light variability observed in *Amborella* are consistent with numerous reports for angiosperm species in both humid tropical forests and temperate forests, as well as in crops and domesticated plants (Buisson and Lee 1993; Miyaji *et al.* 1997; Poorter *et al.* 2006; Lusk *et al.* 2008; Matos *et al.* 2009). Similar leaf responses span both eudicots and monocots (Buisson and Lee 1993; Laurans *et al.* 2012; Yang et al. 2014). Analogous variation in leaf traits has also been described at the intraspecific level in *Arabidopsis thaliana* (Pigliucci and Kolodynska 2002; Poorter *et al.* 2009). Further, similar LMA increases in response to light have been recorded across gymnosperm species (Abrams and Kubiske 1990; Bond *et al.* 1999) and also within individuals (Koch *et al.* 2004). Our documentation of leaf trait variability as a consequence of habitat openings in *Amborella* highlights the adaptive importance of this phenotypic response, which seems to operate in similar ways across the major lineages of vascular plants.

Along with the previously exposed effects of light availability on internode elongation, crown architecture and leaf traits, *Amborella* also provided evidence for shade-induced increases in stem MOE and *EI* (Table 3). This response is consistent with previous analyses describing similar increases in stem stiffness under shaded conditions (Gallenmüller *et al.* 2004; Anten *et al.* 2005; Watari *et al.* 2014; Huber *et al.* 2014). Given that bending moment increases with stature, increases in MOE and *EI* might make shade axes less prone to bending or breakage (Niklas 1992). Maintaining a structural integrity to retain leaves in certain positions might be particularly important under shaded conditions, in which fewer positions provide optimal light interception (Liu *et al.* 2007). Under open canopy conditions, *Amborella* produces stems with greater flexibility (lower MOE) and lower flexural rigidity. Wind speed and drag forces are greater in open canopy habitats (Speck 2003). Therefore, the construction of flexible axes can lead to an increased mechanical resilience, and even large displacements would not affect light interception (Puijalon *et al.* 2011). Wind-induced mechanical stimuli might also explain the production of shorter axes under open canopy. The reduction in shoot elongation is one of the most consistent thigmomorphogenetic effects of mechanical stimulus on plant growth (Badel *et al.* 2015). The coupled reduction of length and stiffness in *Amborella* stems growing under open canopy seem likely functionally beneficial because these characteristics might enable reduced drag forces and easier reconfiguration in wind flow.

### Trait coordination and tradeoffs, different strategies within a single species

The study of key functional traits and their variation across species can be very informative regarding plant ecological strategies (Westoby and Wright 2006). For instance, the median LMA in *Amborella* (74.5 g m^−^^2^, *n* = 409) is very close to the 73 g m^−^^2^ median reported for tropical rainforest species in general (Poorter *et al.*, 2009). The LMA observed here is thus congruent with the habitat preferences of *Amborella*, whose distribution is restricted to rainforest habitats (Poncet *et al.* 2013; Pouteau *et al.* 2015). It is now broadly accepted that plant functional traits need to be studied in a 'network' perspective, with multiple traits correlation and tradeoffs assemblages shaping the ecological strategies of species (Poorter *et al.* 2014). Our results show that coordination of leaf and stem economic traits is present at the intraspecific level in *Amborella*. Hence, *Amborella* traits can shift in a coordinated way as a response to local light environments.

*Amborella* stems growing under open canopies seem to adopt a resource conservation strategy, as suggested by their higher values of LMA and LDMC. The characteristics of leaves in open canopy environments seem to be coordinated with stem traits that reflect similar conservation strategies. Indeed, mass allocation to leaves and stems seems to be coordinated in *Amborella*, as shown by the positive correlations between LDMC/LMA and SSD. A similar positive correlation between LDMC and SSD has been observed across angiosperm species (Méndez-Alonzo *et al.* 2012), suggesting coordinated evolution between these leaf and stem traits. A coordinated increase in mass allocation to stems and leaves may confer a survival advantage in open habitats by reducing the probability of physical hazards (Zimmerman *et al.* 1994; Wright *et al.* 2004; Poorter *et al.* 2009). At the other extreme, closed canopy *Amborella* stems had lower values of leaf and stem mass allocation, which were coupled with higher internode extension. Fast stem extension at internodes can minimize self-shading in large-leaved branches (White 1983). The fast growth of the large-leaved shade axes in *Amborella* is reflected by their longer internodes and their lower values of SSD. It has been shown that wood density, which greatly contributes to SSD, is negatively related with photosynthetic capacity (Santiago *et al.* 2004). Accordingly, efficient acquisition of photosynthates likely allows shade branches to have accelerated volumetric expansion, lowering stem tissue densities.

In addition to positively related traits, we also observed negatively related traits that could indicate tradeoffs. Our study suggests inter-organ tradeoffs in *Amborella* such as the negative relationship between SSD of apical branch sections and LA. Apical SSD explained 27 % of the variation in leaf size in *Amborella* (Fig. 4), which is similar to the relationships presented by Wright *et al.* (2007) when relating LA and wood density of simple-leaved species. The apical SSD-LA tradeoff in *Amborella* is consistent with similar findings of negative relations of leaf size with both wood density and branch mechanical stiffness across species of different habitats (Pickup *et al.* 2005; Wright *et al.* 2006; Wright *et al.* 2007; Swenson and Enquist 2008; Olson *et al.* 2009). To our knowledge, this is the first evidence of this tradeoff among individuals within a species. Wright *et al.* (2006; 2007) explained this tradeoff via plant hydraulics, suggesting that stems with low wood density enable higher hydraulic conductivity per sapwood area (K_S_), allowing higher leaf surface. However, a previous study (Feild *et al.* 2001) has shown that K_S_ was not different between sun-exposed and understory branches of *Amborella*, which, as we have shown here, tend to have significant variation in LA. Therefore, the accelerated growth of wide-leaved closed canopy axes, which can be regarded as a common shade avoidance syndrome (Schmitt *et al.* 2003), might underlie the apical SSD-LA tradeoff observed in *Amborella*.

Another tradeoff is likely indicated by the strong negative relationship between SWC and SSD. This pattern may emerge as a compromise between mechanical strength and water storage (Santiago *et al.* 2004). Our data were consistent with such a tradeoff in *Amborella*, with the observed negative relationship between both stem mechanical traits (MOR and MOE) and SWC. Respectively, gains in mechanical strength were observed as SSD increases. These results are congruent with studies showing that lower density is associated with lower capacity to resist bending and breakage, but higher xylem water conductivity and storage (Pratt *et al.* 2007; Onoda *et al.* 2010; Méndez-Alonzo *et al.* 2012). Rosell *et al.* (2012) proposed a possible mechanism for this tradeoff, suggesting that higher levels of stem water storage would be associated with greater allocation to the cell lumen and less to the cell walls that are largely responsible for stem material mechanical stiffness.

### *Amborella* architecture and mechanical properties in the context of angiosperm growth form evolution

Flowering plants have evolved an unparalleled diversity of growth forms and architectures. The architecture of a plant is defined by the nature and arrangement of each of its parts (Barthélémy and Caraglio 2007). Because most of the axes of *Amborella* are initially orthotropic, bending secondarily by gravity, its architecture corresponds to Champagnat's architectural model (Hallé *et al.* 1978). However, the observed changes in leaf orientation, from radial to bilateral symmetry according to their position on the axis, are features of Mangenot's architectural model (Hallé *et al.* 1978). *Amborella* may thus represent an intermediate form suggesting a continuum between these two architectural models. Both of the previously cited models are often characterized by the lack of a main trunk and construction based on the superposition of modules. Such repeated growth from lateral meristems is known as sympodiality, which has been suggested as a synapomorphy for the angiosperms (Carlquist 2009).

Sympodiality, which is widespread among basal angiosperms, has also been suggested as a retained character in Ranunculales, the eudicot order sister to the rest of the eudicots (APG, 2016). Because of this phylogenetic distribution and because of the general absence of sympodial growth in gymnosperms, it seems possible that the angiosperm ancestor was sympodial. Carlquist (2009) suggested that sympodiality may have provided angiosperms with numerous competitive advantages, such as rapid spreading over wider lateral areas, securing footholds and tapping new soil resources by the rooting of branches, and escaping hydraulic and mechanical failures by the production of numerous branches that can potentially root. Additionally, the production of branches from dormant buds confers on *Amborella* the ability to resprout through basitonic and mesotonic relays (Fig. 1E and F). Resprouters seem to have an increased ability to persist after disturbance events (Bond and Midgley 2001). As such, sprouting ability has been suggested to be a key feature of plant strategies (Bond and Midgley 2001). Collar sprouting, as seen in the basitonic relays observed in *Amborella* (Fig. 1F), is generally rare in conifers (Del Tredici 2001). The sprouting ability and sympodial construction observed in *Amborella* are morphological characteristics observed in other cane-like representatives of basal angiosperm groups (Isnard *et al.*, 2012). This suggests that sympodiality, along with sprouting and rooting ability, which can be considered as competitive morphological attributes, were acquired early during the evolution of the flowering plants.

The cane-like form of *Amborella* and other basal angiosperms is often associated with the presence of scandent stems (Feild and Arens 2007). The laxity of these scandent stems should be reflected by the relationship between stem size and stem mechanical properties. The vesselless stems of *Amborella* show the mechanical organization of a self-supporting plant with stem material stiffness increasing with stem diameter (Fig. 6A; Table 3). A previous study analyzing the stem mechanics of cane-like basal angiosperms highlighted a similar mechanical organization in shrubs within *Aristolochia*, *Thottea*, and Piperaceae (Isnard *et al.* 2012). This suggests that mechanical reinforcement does not compensate for increasing plant stature in cane-like species. It has been suggested that variations in stem geometry are sufficient to generate functional and morphological diversity even in the absence of shifts in stem material properties (Gartner 1991; Rosell *et al.* 2012). Therefore, the scandent form of *Amborella* and other cane-like basal angiosperms likely evolves readily as the result of increases in SL without an offset in stem diameter.

## Conclusions

Our architectural description shows that *Amborella* growth involves the stacking of sympodial modules. The axes making up these sympodial modules have a pattern of increasing mechanical stiffness with increasing diameter, corresponding to the mechanical profile of a self-supporting plant. The sympodial growth observed in *Amborella*, which is associated with numerous competitive advantages, might be a synapomorphy for the angiosperms. Canopy openness triggered changes in whole-plant architecture, varying from a long-branched shrub with pendulous axes under closed canopy versus a short- and densely branched self-supporting shrub under open canopy. Further, canopy openness significantly influenced leaf size and leaf mass investment. However, our analyses show that across light environments, LA and stem size predict one another, following Corner’s Rules, with stem tissue density negatively correlated with LA. The documentation of this coordination of traits in the sister to all other flowering plants, reaffirms the pervasiveness of these trait constellations. Trait coordination along the leaf and stem economic spectra likely provides *Amborella* with adaptive functional strategies under variation in canopy openness. The phylogenetic distribution of these responses suggests that similar plastic responses to light availability were plausibly present in the common ancestor of all living angiosperms. 

## References

[plw068-B1] Abrams MD Kubiske ME. 1990 Leaf structural characteristics of 31 hardwood and conifer tree species in central Wisconsin: Influence of light regime and shade-tolerance rank. Forest Ecology and Management 31:245–253.

[plw068-B2] Ackerly DD Donoghue MJ. 1998 Leaf size, sapling allometry, and Corner's Rules: phylogeny and correlated evolution in Maples (*Acer*). The American Naturalist 152:767–791. 10.1086/286208 18811427

[plw068-B3] Amborella Genome Project 2013 The *Amborella* genome and the evolution of flowering plants. Science 342:1241089. 24357323 10.1126/science.1241089

[plw068-B4] Anten NPR Casado-Garcia R Nagashima H. 2005 Effects of mechanical stress and plant density on mechanical characteristics, growth, and lifetime reproduction of tobacco plants. The American Naturalist 166:650–660. 10.1086/497442 16475082

[plw068-B5] APG 2016 An update of the Angiosperm Phylogeny Group classification for the orders and families of flowering plants: APG IV. Botanical Journal of the Linnean Society 181:1–20.

[plw068-B6] Badel E Ewers F Cochard H Telewski FW. 2015 Acclimation of mechanical and hydraulic functions in trees: Impact of the thigmomorphogenetic process. Frontiers in Plant Science 6. 10.3389/fpls.2015.00266 PMC4406077 25954292

[plw068-B7] Barthélémy D Caraglio Y. 2007 Plant architecture: a dynamic, multilevel and comprehensive approach to plant form, structure and ontogeny. Annals of Botany 99:375–407. 17218346 10.1093/aob/mcl260 PMC2802949

[plw068-B8] Bond BJ Farnsworth BT Coulombe RA Winner WE. 1999 Foliage physiology and biochemistry in response to light gradients in conifers with varying shade tolerance. Oecologia 120:183–192. 10.1007/s004420050847 28308078

[plw068-B9] Bond WJ Midgley JJ. 2001 Ecology of sprouting in woody plants: the persistence niche. Trends in Ecology & Evolution 16:45–51. 11146144 10.1016/s0169-5347(00)02033-4

[plw068-B10] Buisson D Lee DW. 1993 The developmental responses of Papaya leaves to simulated canopy shade. American Journal of Botany 80:947–952.

[plw068-B11] Carlquist S. 1996 Wood anatomy of primitive angiosperms: new perspectives and syntheses In: Taylor DW Hickey LJ, eds. Flowering plant origin, evolution & phylogeny. Berlin: Springer, 68–90.

[plw068-B12] Carlquist S. 2001 Observations on the vegetative anatomy of Austrobaileya: habital, organographic and phylogenetic conclusions. Botanical Journal of the Linnean Society 135:1–11.

[plw068-B13] Carlquist S. 2009 Xylem heterochrony: an unappreciated key to angiosperm origin and diversifications. Botanical Journal of the Linnean Society 161:26–65.

[plw068-B14] Castro-Díez P Puyravaud JP Cornelissen JHC. 2000 Leaf structure and anatomy as related to leaf mass per area variation in seedlings of a wide range of woody plant species and types. Oecologia 124:476–486. 10.1007/PL00008873 28308386

[plw068-B15] Chambel M Climent J Alía R Valladares F. 2005 Phenotypic plasticity: a useful framework for understanding adaptation in forest species. Investigación Agraria Sistemas Y Recursos Forestales 14:334–344.

[plw068-B16] Charles-Dominique T Edelin C Bouchard A. 2010 Architectural strategies of *Cornus sericea*, a native but invasive shrub of southern Quebec, Canada, under an open or a closed canopy. Annals of Botany 105:205–220. 19900945 10.1093/aob/mcp273 PMC2814749

[plw068-B17] Charles-Dominique T Edelin C Brisson J Bouchard A. 2012 Architectural strategies of *Rhamnus cathartica* (Rhamnaceae) in relation to canopy openness. Botany 90:976–989.

[plw068-B18] Charles-Dominique T Edelin C Bouchard A Legendre P Brisson J. 2015 Using intra-individual variation in shrub architecture to explain population cover. Oikos 124:707–716.

[plw068-B19] Cornelissen JHC. 1993 Aboveground morphology of shade-tolerant *Castanopsis fargesii* saplings in response to light environment. International Journal of Plant Sciences 154:481–495.

[plw068-B20] Cornelissen JHC. 1999 A triangular relationship between leaf size and seed size among woody species: allometry, ontogeny, ecology and taxonomy. Oecologia 118:248–255. 10.1007/s004420050725 28307701

[plw068-B21] Corner E. 1949 The durian theory or the origin of the modern tree. Annals of Botany 13:367–414.

[plw068-B22] Crisp MD Cook LG. 2005 Do early branching lineages signify ancestral traits?. Trends in Ecology & Evolution 20:122–128. 16701355 10.1016/j.tree.2004.11.010

[plw068-B23] Del Tredici P. 2001 Sprouting in temperate trees: a morphological and ecological review. The Botanical Review 67:121–140.

[plw068-B24] Díaz S Kattge J Cornelissen JHC Wright IJ Lavorel S Dray S Reu B Kleyer M Wirth C Colin Prentice I, 2016 The global spectrum of plant form and function. Nature 529:167–171. 26700811 10.1038/nature16489

[plw068-B25] Enquist BJ. 2002 Universal scaling in tree and vascular plant allometry: toward a general quantitative theory linking plant form and function from cells to ecosystems. Tree Physiology 22:1045–1064. 12414366 10.1093/treephys/22.15-16.1045

[plw068-B26] Feild TS Arens NC. 2005 Form, function and environments of the early angiosperms: merging extant phylogeny and ecophysiology with fossils. New Phytologist 166:383–408. 15819904 10.1111/j.1469-8137.2005.01333.x

[plw068-B27] Feild TS Arens NC. 2007 The ecophysiology of early angiosperms. Plant, Cell & Environment 30:291–309. 10.1111/j.1365-3040.2006.01625.x 17263775

[plw068-B28] Feild TS Brodribb T Jaffré T Holbrook NM. 2001 Acclimation of leaf anatomy, photosynthetic light use, and xylem hydraulics to light in *Amborella trichopoda* (Amborellaceae). International Journal of Plant Sciences 162:999–1008.

[plw068-B29] Feild TS Wilson JP. 2012 Evolutionary voyage of angiosperm vessel structure-function and its significance for early angiosperm success. International Journal of Plant Sciences 173:596–609.

[plw068-B30] Feild TS Zweiniecki MA Brodribb T Jaffré T Donoghue MJ Holbrook NM. 2000 Structure and function of tracheary elements in *Amborella trichopoda*. International Journal of Plant Sciences 161:705–712.

[plw068-B31] Frazer GW Canham C Lertzman K. 1999 Gap light analyzer (GLA), version 2.0: imaging software to extract canopy structure and gap light transmission indices from true-colour fisheye photographs, users manual and program documentation. Simon Fraser University, Burnaby, British Columbia, and the Institute of Ecosystem Studies, Millbrook, New York.

[plw068-B32] Fusco G Minelli A. 2010 Phenotypic plasticity in development and evolution: facts and concepts. Philosophical Transactions of the Royal Society of London B: Biological Sciences 365:547–556. 20083631 10.1098/rstb.2009.0267 PMC2817147

[plw068-B33] Gallenmüller F Rowe N Speck T. 2004 Development and growth form of the neotropical Liana *Croton nuntians*: the effect of light and mode of attachment on the biomechanics of the stem. Journal of Plant Growth Regulation 23:83–97.

[plw068-B34] Gartner BL. 1991 Structural stability and architecture of vines vs. shrubs of poison oak, *Toxicodendron diversilobum*. Ecology 72:2005–2015. 10.1007/BF00325255 28313834

[plw068-B35] Gere JM Timoshenko SP. 1999 Mechanics of materials. Cheltenham, UK: Stanley Thornes.

[plw068-B36] Hallé F Oldeman RA Tomlinson PB. 1978 Tropical trees and forests. Berlin: Springer-Verlag.

[plw068-B37] Harvey PH Pagel MD. 1991 The comparative method in evolutionary biology. Oxford: Oxford University Press.

[plw068-B38] Huber H De Brouwer J Von Wettberg EJ During HJ Anten NPR. 2014 More cells, bigger cells or simply reorganization? Alternative mechanisms leading to changed internode architecture under contrasting stress regimes. New Phytologist 201:193–204. 24033342 10.1111/nph.12474

[plw068-B39] Isnard S Feild TS. 2015 The evolution of angiosperm lianescence: a perspective from xylem structure-function In: Schnitzer SA Bongers F Burnham RJ Putz FE, eds. Ecology of Lianas. Oxford, United Kingdom: John Wiley & Sons, Ltd, 221–238.

[plw068-B40] Isnard S Prosperi J Wanke S Wagner S Samain M-S Trueba S Frenzke L Neinhuis C Rowe NP. 2012 Growth form evolution in Piperales and its relevance for understanding the angiosperm diversification - an integrative approach combining plant architecture, anatomy and biomechanics. International Journal of Plant Sciences 173:610–639.

[plw068-B41] Jennings S Brown N Sheil D. 1999 Assessing forest canopies and understorey illumination: canopy closure, canopy cover and other measures. Forestry 72:59–74.

[plw068-B42] Jérémie J. 1982 *Monimiacées, Amborellacées, Atherospermatacées, Trimeniacées, Chloranthaceae.* Flore de la nouvelle-calédonie et dépendances. Paris, France: Muséum National d'Histoire Naturelle.

[plw068-B43] Kawamura K Takeda H. 2002 Light environment and crown architecture of two temperate Vaccinium species: inherent growth rules versus degree of plasticity in light response. Canadian Journal of Botany 80:1063–1077.

[plw068-B44] Koch GW Sillett SC Jennings GM Davis SD. 2004 The limits to tree height. Nature 428:851–854. 15103376 10.1038/nature02417

[plw068-B45] Lahaye R Civeyrel L Speck T Rowe NP. 2005 Evolution of shrub-like growth forms in the lianoid subfamily Secamonoideae (Apocynaceae s.l.) of Madagascar: phylogeny, biomechanics, and development. American Journal of Botany 92:1381–1396. 21646158 10.3732/ajb.92.8.1381

[plw068-B46] Laurans M Martin O Nicolini E Vincent G. 2012 Functional traits and their plasticity predict tropical trees regeneration niche even among species with intermediate light requirements. Journal of Ecology 100:1440–1452.

[plw068-B106] Liu Y Schieving F Stuefer JF Anten NPR. 2007 The effects of mechanical stress and spectral shading on the growth and allocation of ten genotypes of a stoloniferous plant. Annals of Botany 99:121–130. 17085473 10.1093/aob/mcl230 PMC2802973

[plw068-B47] Losos JB. 2011 Convergence, adaptation, and constraint. Evolution 65:1827–1840. 21729041 10.1111/j.1558-5646.2011.01289.x

[plw068-B48] Lusk CH Reich PB Montgomery RA Ackerly DD Cavender-Bares J. 2008 Why are evergreen leaves so contrary about shade? Trends in Ecology & Evolution 23:299–303. 18439708 10.1016/j.tree.2008.02.006

[plw068-B49] Mathews S Donoghue MJ. 1999 The root of angiosperm phylogeny inferred from duplicate phytochrome genes. Science 286:947–950. 10542147 10.1126/science.286.5441.947

[plw068-B50] Mathews S Donoghue MJ. 2000 Basal angiosperm phylogeny inferred from duplicate phytochromes A and C. International Journal of Plant Sciences 161:S41–S55. 10.1126/science.286.5441.947 10542147

[plw068-B51] Matos FS Wolfgramm R Gonçalves FV Cavatte PC Ventrella MC Damatta FM. 2009 Phenotypic plasticity in response to light in the coffee tree. Environmental and Experimental Botany 67:421–427.

[plw068-B52] Méndez-Alonzo R Paz H Zuluaga RC Rosell JA Olson ME. 2012 Coordinated evolution of leaf and stem economics in tropical dry forest trees. Ecology 93:2397–2406. 23236911 10.1890/11-1213.1

[plw068-B53] Miyaji K-I Da Silva WS Alvim PDT. 1997 Productivity of leaves of a tropical tree, Theobroma cacao, grown under shading, in relation to leaf age and light conditions within the canopy. New Phytologist 137:463–472. 10.1046/j.1469-8137.1997.00841.x 33863072

[plw068-B54] Niinemets Ü. 1996 Changes in foliage distribution with relative irradiance and tree size: differences between the saplings of *Acer platanoides* and *Quercus robur*. Ecological Research 11:269–281.

[plw068-B55] Niinemets Ü Kull O Tenhunen JD. 1999 Variability in leaf morphology and chemical composition as a function of canopy light environment in coexisting deciduous trees. International Journal of Plant Sciences 160:837–848. 10506464 10.1086/314180

[plw068-B56] Niklas KJ. 1992 Plant biomechanics: an engineering approach to plant form and function. Chicago: University of Chicago Press.

[plw068-B57] Niklas KJ Enquist BJ. 2002 Canonical rules for plant organ biomass partitioning and annual allocation. American Journal of Botany 89:812–819. 21665681 10.3732/ajb.89.5.812

[plw068-B58] Nixon KC Wheeler QD. 1990 An amplification of the phylogenetic species concept. Cladistics 6:211–223.

[plw068-B59] Olson ME Aguirre-Hernández R Rosell JA. 2009 Universal foliage-stem scaling across environments and species in dicot trees: plasticity, biomechanics and Corner’s Rules. Ecology Letters 12:210–219. 19141123 10.1111/j.1461-0248.2008.01275.x

[plw068-B60] Onoda Y Richards AE Westoby M. 2010 The relationship between stem biomechanics and wood density is modified by rainfall in 32 Australian woody plant species. New Phytologist 185:493–501. 19925557 10.1111/j.1469-8137.2009.03088.x

[plw068-B61] Parkhurst DF Loucks OL. 1972 Optimal Leaf Size in Relation to Environment. Journal of Ecology 60:505–537.

[plw068-B62] Pérez-Harguindeguy N Díaz S Garnier E Lavorel S Poorter H Jaureguiberry P Bret-Harte MS Cornwell WK Craine JM Gurvich DE, 2013 New handbook for standardised measurement of plant functional traits worldwide. Australian Journal of Botany 61:167–234.

[plw068-B63] Pickup M Westoby M Basden A. 2005 Dry mass costs of deploying leaf area in relation to leaf size. Functional Ecology 19:88–97.

[plw068-B64] Pigliucci M Kolodynska A. 2002 Phenotypic plasticity to light intensity in *Arabidopsis thaliana*: invariance of reaction norms and phenotypic integration. Evolutionary Ecology 16:27–47.

[plw068-B65] Pigliucci M Murren CJ Schlichting CD. 2006 Phenotypic plasticity and evolution by genetic assimilation. Journal of Experimental Biology 209:2362–2367. 16731812 10.1242/jeb.02070

[plw068-B66] Pineda-García F Paz H Meinzer FC. 2013 Drought resistance in early and late secondary successional species from a tropical dry forest: the interplay between xylem resistance to embolism, sapwood water storage and leaf shedding. Plant, Cell & Environment 36:405–418. 10.1111/j.1365-3040.2012.02582.x 22812458

[plw068-B67] Poncet V Munoz F Munzinger J Pillon Y Gomez C Couderc M Tranchant-Dubreuil C Hamon S De Kochko A. 2013 Phylogeography and niche modelling of the relict plant Amborella trichopoda (Amborellaceae) reveal multiple Pleistocene refugia in New Caledonia. Molecular Ecology 22:6163–6178. 24118476 10.1111/mec.12554

[plw068-B68] Poorter L. 1999 Growth responses of 15 rain-forest tree species to a light gradient: the relative importance of morphological and physiological traits. Functional Ecology 13:396–410.

[plw068-B69] Poorter H Lambers H Evans JR. 2014 Trait correlation networks: a whole-plant perspective on the recently criticized leaf economic spectrum. New Phytologist 201:378–382. 24117716 10.1111/nph.12547

[plw068-B70] Poorter L Mcdonald I Alarcón A Fichtler E Licona J-C Peña-Claros M Sterck F Villegas Z Sass-Klaassen U. 2010 The importance of wood traits and hydraulic conductance for the performance and life history strategies of 42 rainforest tree species. New Phytologist 185:481–492. 19925555 10.1111/j.1469-8137.2009.03092.x

[plw068-B71] Poorter H Niinemets Ü Poorter L Wright IJ Villar R. 2009 Causes and consequences of variation in leaf mass per area (LMA): a meta-analysis. New Phytologist 182:565–588. 19434804 10.1111/j.1469-8137.2009.02830.x

[plw068-B72] Poorter H Niklas KJ Reich PB Oleksyn J Poot P Mommer L. 2012 Biomass allocation to leaves, stems and roots: meta-analyses of interspecific variation and environmental control. New Phytologist 193:30–50. 22085245 10.1111/j.1469-8137.2011.03952.x

[plw068-B73] Poorter H Pepin S Rijkers T De Jong Y Evans JR Körner C. 2006 Construction costs, chemical composition and payback time of high- and low-irradiance leaves. Journal of Experimental Botany 57:355–371. 16303828 10.1093/jxb/erj002

[plw068-B74] Pouteau R Trueba S Feild TS Isnard S. 2015 New Caledonia: a Pleistocene refugium for rain forest lineages of relict angiosperms. Journal of Biogeography 42:2062–2077.

[plw068-B75] Pratt RB Jacobsen AL Ewers FW Davis SD. 2007 Relationships among xylem transport, biomechanics and storage in stems and roots of nine Rhamnaceae species of the California chaparral. New Phytologist 174:787–798. 17504462 10.1111/j.1469-8137.2007.02061.x

[plw068-B76] Puijalon S Bouma TJ Douady CJ Van Groenendael J Anten NPR Martel E Bornette G. 2011 Plant resistance to mechanical stress: evidence of an avoidance–tolerance trade-off. New Phytologist 191:1141–1149. 21585390 10.1111/j.1469-8137.2011.03763.x

[plw068-B77] Qiu YL Lee J Bernasconi‐Quadroni F Soltis DE Soltis PS Zanis M Zimmer EA Chen Z Savolainen V Chase MW. 2000 Phylogeny of basal angiosperms: analyses of five genes from three genomes. International Journal of Plant Sciences 161:S3–S27.

[plw068-B78] Rosell JA Olson ME Aguirre-Hernández R Sánchez-Sesma FJ. 2012 Ontogenetic modulation of branch size, shape, and biomechanics produces diversity across habitats in the Bursera simaruba clade of tropical trees. Evolution & Development 14:437–449. 22947317 10.1111/j.1525-142X.2012.00564.x

[plw068-B79] Rowe N Speck T. 2005 Plant growth forms: an ecological and evolutionary perspective. New Phytologist 166:61–72. 15760351 10.1111/j.1469-8137.2004.01309.x

[plw068-B80] Rozendaal DMA Hurtado VH Poorter L. 2006 Plasticity in leaf traits of 38 tropical tree species in response to light; relationships with light demand and adult stature. Functional Ecology 20:207–216.

[plw068-B81] Santiago LS Goldstein G Meinzer FC Fisher JB Machado K Woodruff D Jones T. 2004 Leaf photosynthetic traits scale with hydraulic conductivity and wood density in Panamanian forest canopy trees. Oecologia 140:543–550. 15232729 10.1007/s00442-004-1624-1

[plw068-B82] Schmitt J Stinchcombe JR Heschel MS Huber H. 2003 The Adaptive Evolution of Plasticity: Phytochrome-Mediated Shade Avoidance Responses. Integrative and Comparative Biology 43:459–469. 21680454 10.1093/icb/43.3.459

[plw068-B83] Scotland RW. 2011 What is parallelism?. Evolution & Development 13:214–227. 21410877 10.1111/j.1525-142X.2011.00471.x

[plw068-B84] Smith RJ. 2009 Use and misuse of the reduced major axis for line‐fitting. American Journal of Physical Anthropology 140:476–486. 19425097 10.1002/ajpa.21090

[plw068-B85] Soltis DE Soltis PS Chase MW Mort ME Albach DC Zanis M Savolainen V Hahn WH Hoot SB Fay MF, 2000 Angiosperm phylogeny inferred from 18S rDNA, rbcL, and atpB sequences. Botanical Journal of the Linnean Society 133:381–461.

[plw068-B86] Soltis PS Soltis DE Chase MW. 1999 Angiosperm phylogeny inferred from multiple genes as a tool for comparative biology. Nature 402:402–404. 10586878 10.1038/46528

[plw068-B87] Speck O. 2003 Field measurements of wind speed and reconfiguration in Arundo donax (Poaceae) with estimates of drag forces. American Journal of Botany 90:1253–1256. 21659225 10.3732/ajb.90.8.1253

[plw068-B88] Sperry JS Hacke UG Feild TS Sano Y Sikkema EH. 2007 Hydraulic consequences of vessel evolution in angiosperms. International Journal of Plant Sciences 168:1127–1139.

[plw068-B89] Sultan SE. 2000 Phenotypic plasticity for plant development, function and life history. Trends in Plant Science 5:537–542. 11120476 10.1016/s1360-1385(00)01797-0

[plw068-B90] Sun S Jin D Shi P. 2006 The leaf size – twig size spectrum of temperate woody species along an altitudinal gradient: an invariant allometric scaling relationship. Annals of Botany 97:97–107. 16254019 10.1093/aob/mcj004 PMC2803375

[plw068-B91] Swenson NG Enquist BJ. 2008 The relationship between stem and branch wood specific gravity and the ability of each measure to predict leaf area. American Journal of Botany 95:516–519. 21632377 10.3732/ajb.95.4.516

[plw068-B92] Thien LB Sage TL Jaffré T Bernhardt P Pontieri V Weston PH Malloch D Azuma H Graham SW Mcpherson MA, 2003 The population structure and floral biology of *Amborella trichopoda* (Amborellaceae). Annals of the Missouri Botanical Garden 90:466–490.

[plw068-B93] Valladares F Niinemets Ü. 2008 Shade tolerance, a key plant feature of complex nature and consequences. Annual Review of Ecology, Evolution, and Systematics 39:237.

[plw068-B94] Villar R Ruiz-Robleto J Ubera JL Poorter H. 2013 Exploring variation in leaf mass per area (LMA) from leaf to cell: an anatomical analysis of 26 woody species. American Journal of Botany 100:1969–1980. 24107583 10.3732/ajb.1200562

[plw068-B95] Warton DI Duursma RA Falster DS Taskinen S. 2012 smatr 3– an R package for estimation and inference about allometric lines. Methods in Ecology and Evolution 3:257–259.

[plw068-B96] Watari R Nagashima H Hirose T. 2014 Stem extension and mechanical stability of Xanthium canadense grown in an open or in a dense stand. Annals of Botany 114:179–190. 24879768 10.1093/aob/mcu088 PMC4071106

[plw068-B97] Westoby M Falster DS Moles AT Vesk PA Wright IJ. 2002 Plant ecological strategies: some leading dimensions of variation between species. Annual Review of Ecology and Systematics 33:125–159.

[plw068-B98] Westoby M Wright I. 2003 The leaf size – twig size spectrum and its relationship to other important spectra of variation among species. Oecologia 135:621–628. 16228258 10.1007/s00442-003-1231-6

[plw068-B99] Westoby M Wright IJ. 2006 Land-plant ecology on the basis of functional traits. Trends in Ecology & Evolution 21:261–268. 16697912 10.1016/j.tree.2006.02.004

[plw068-B100] White PS. 1983 Evidence that temperate east north American evergreen woody plants follow Corner's Rules. New Phytologist 95:139–145.

[plw068-B101] Wright IJ Reich PB Westoby M Ackerly DD Baruch Z Bongers F Cavender-Bares J Chapin T Cornelissen JH Diemer M. 2004 The worldwide leaf economics spectrum. Nature 428:821–827. 15103368 10.1038/nature02403

[plw068-B102] Wright IJ Ackerly DD Bongers F Harms KE Ibarra-Manriquez G Martinez-Ramos M Mazer SJ Muller-Landau HC Paz H Pitman NCA, 2007 Relationships among ecologically important dimensions of plant trait variation in seven neotropical forests. Annals of Botany 99:1003–1015. 16595553 10.1093/aob/mcl066 PMC2802905

[plw068-B103] Wright IJ Falster DS Pickup M Westoby M. 2006 Cross-species patterns in the coordination between leaf and stem traits, and their implications for plant hydraulics. Physiologia Plantarum 127:445–456.

[plw068-B104] Yang S-J Sun M Zhang Y-J Cochard H Cao K-F. 2014 Strong leaf morphological, anatomical, and physiological responses of a subtropical woody bamboo (*Sinarundinaria nitida*) to contrasting light environments. Plant Ecology 215:97–109.

[plw068-B105] Zimmerman JK Iii EME Waide RB Lodge DJ Taylor CM Brokaw NVL. 1994 Responses of Tree Species to Hurricane Winds in Subtropical Wet Forest in Puerto Rico: Implications for Tropical Tree Life Histories. Journal of Ecology 82:911–922.

